# Dental pulp stem cell-derived exosomes revitalize salivary gland epithelial cell function in NOD mice via the GPER-mediated cAMP/PKA/CREB signaling pathway

**DOI:** 10.1186/s12967-023-04198-0

**Published:** 2023-06-03

**Authors:** Shilin Hu, Bo Chen, Jiannan Zhou, Fangqi Liu, Tianjiao Mao, Janak L. Pathak, Nobumoto Watanabe, Jiang Li

**Affiliations:** 1 Guangdong Engineering Research Center of Oral Restoration and Reconstruction, Affiliated Stomatology Hospital of Guangzhou Medical University, #195 Dongfeng West Road, Guangzhou, 510140 Guangdong China; 2 Chemical Biology Research Group, RIKEN Center for Sustainable Resource Science, Wako, Saitama 351-0198 Japan; 3 Bio-Active Compounds Discovery Unit, RIKEN Center for Sustainable Resource Science, Wako, Saitama 351-0198 Japan

**Keywords:** Sjogren’s syndrome (SS), Salivary gland epithelial cells (SGEC), Dental pulp stem cells (DPSC), Exosomes, Aquaporin 5 (AQP5), G-protein coupled estrogen receptor (GPER)

## Abstract

**Background:**

Restoration of salivary gland function in Sjogren’s syndrome (SS) is still a challenge. Dental pulp stem cells (DPSCs) derived exosomes had shown anti-inflammatory, anti-oxidative, immunomodulatory, and tissue function restorative abilities. However, the salivary gland function restoration potential of DPSCs-derived exosomes (DPSC-Exos) during SS has not been investigated yet.

**Methods:**

DPSC-Exos was isolated by ultracentrifugation methods and characterized. Salivary gland epithelial cells (SGEC) were treated with interferon-gamma (IFN-γ) to mimic SS in vitro and cultured with or without DPSC-Exos. SGEC survival and aquaporin 5 (AQP5) expression were analyzed. mRNA sequencing and bioinformatics analysis were performed in IFN-γ vs. DPSC-Exos+ IFN-γ treated SGEC. Non-obese diabetic (NOD)/ltj female mice (SS model), were intravenously administered with DPSC-Exos, and salivary gland functions and SS pathogenicity were analyzed. Furthermore, the mRNA sequencing and bioinformatics predicted mechanism of the therapeutic effect of DPSC-Exos was further investigated both in vitro and in vivo using RT-qPCR, Western blot, immunohistochemistry, immunofluorescence, flowcytometry analysis.

**Results:**

DPSC-Exos partially rescued IFN-γ triggered SGEC death. IFN-γ inhibited AQP5 expression in SGEC and DPSC-Exos reversed this effect. Transcriptome analysis showed GPER was the upregulated DEG in DPSC-Exos-treated SGEC with a positive correlation with salivary secretion-related DEGs. Pathway enrichment analysis revealed that DEGs were mainly attributed to estrogen 16 alpha-hydroxylase activity, extracellular exosome function, cAMP signaling, salivary secretion, and estrogen signaling. Intravenous injection of DPSC-Exos in NOD/ltj mice alleviated the SS syndrome as indicated by the increased salivary flow rate, attenuated glandular inflammation, and increased AQP5 expression. GPER was also upregulated in the salivary gland of DPSC-Exos-treated NOD/ltj mice compared with the PBS-treated NOD/ltj mice. IFN-γ+DPSC-Exos-treated SGEC showed higher expression of AQP5, p-PKA, cAMP, and intracellular Ca^2+^ levels compared with IFN-γ-treated SGEC. These effects were reversed by the inhibition of GPER.

**Conclusions:**

Our results showed that DPSC-Exos revitalize salivary gland epithelial cell function during SS via the GPER-mediated cAMP/PKA/CREB pathway suggesting the possible therapeutic potential of DPSC-Exos in SS-treatment.

**Supplementary Information:**

The online version contains supplementary material available at 10.1186/s12967-023-04198-0.

## Background

Sjogren’s syndrome (SS) is a chronic, systemic autoimmune disorder characterized by xerostomia and xerophthalmia that mainly occurred in menopausal women. Xerostomia is a common clinical condition caused by hyposalivation that leads to dysphagia, abnormal taste, dental caries, periodontal disease, and poor quality of life [1]. With the development of the disease, about 2/3 of SS patients have systemic immune dysfunction [2, 3]. There is no gold standard for xerostomia and hyposalivation treatment. Xerostomia treatment is mainly classified into symptomatic, topical, or systemic stimulants and regenerative [4]. The most commonly used symptomatic approaches are increased fluid intake and the use of salivary substitutes which have only a short time effect. Gene therapy [5] still stays in phase I trial and mesenchymal stem cells (MSCs) therapy [6] causes the risk of ectopic osteogenesis and tumorigenesis [7–9] In general, effective treatment approaches for xerostomia are in high demand in clinics.

Exosomes are a type of extracellular vesicle with a size range of 40–150 nm in diameter. Exosomes carry various biological molecules, including proteins, lipids, and RNAs which can be transferred to the recipient cells [10, 11]. It has been reported that the effects of MSCs-derived exosomes (MSC-Exos) contribute to cell-to-cell communication [12]. MSCs-Exos have shown the potential to treat various inflammatory diseases including SS [13–15]. Dental pulp stem cells (DPSCs) are a type of MSCs isolated from dental pulp tissue. DPSCs are highly proliferative and less immunogenic compared to MSCs derived from other sources [16]. Over the past few years, researchers are using DPSCs-derived exosomes (DPSC-Exos) mainly for their anti-apoptotic, anti-inflammatory, and regenerative properties for treating systemic diseases [17, 18]. DPSC-Exos prevents neuronal apoptosis related to the endogenous anti-apoptotic factor Bcl-2 [19]. DPSC-Exos significantly alleviates artificially induced acute inflammation and reduced tissue edema in mice [20]. DPSCs-conditioned media has been reported to attenuate SS in the submandibular glands [21]. However, the therapeutic effects of DPSC-Exos on the alleviation of xerostomia and restoration of salivary gland function in SS have not been investigated yet. Aquaporin 5 (AQP5), a transmembrane channel protein, is mainly expressed in salivary gland epithelial cells (SGEC) and facilitates saliva secretion [22, 23]. The inflammatory condition in SS salivary gland causes SGEC death and the downregulation of AQP5 expression resulting in hyposalivation and xerostomia. Various therapeutic approaches have shown the potential to upregulate AQP5 expression SGEC during SS treatment [24, 25]. However, the effect of DPSC-Exos on SGEC survival and AQP5 expression during SS is still unclear.

This study aims to investigate the therapeutic effect of DPSC-Exos for SS-related xerostomia and the underlying mechanisms. DPSC-Exos were isolated from DPSC cell-sheet culture conditioned medium, characterized, and used for in vitro and in vivo studies. A253 cell line was used as a model of SGEC [26]. The SGEC was treated with interferon-gamma (IFN-γ) to mimic the inflammatory condition of SS. Non-obese diabetic (NOD)/ltj female mice were used as a SS animal model.

## Materials and methods

### Isolation, culture, and characterization of human DPSCs

#### Isolation of human DPSCs

This study was approved by the institutional review board of the Affiliated Stomatology Hospital of Guangzhou Medical University (JCYJ2022020). Dental pulp tissues were dissected and pooled from teenagers under 18-year-old premolar (n = 10, male). Briefly, pulp tissue was washed with phosphate buffer saline (PBS). Then, the tissue was cut into small pieces using ophthalmic scissors, placed in cell culture flasks, and the extracellular matrix was digested with Dispase II (4 mg/ml), Collagenase I (3 mg/ml) (Sigma Aldrich, St. Louis, MO, USA) supplemented for 40 min at 37 °C. After centrifugation, the tissue was maintained in alpha minimum essential Eagle’s medium (α-MEM) (Gibco, Waltham, MA, USA) supplemented with 10% fetal bovine serum (FBS) (Gibco, Waltham, MA, USA) and 100 U/ml penicillin/streptomycin (Gibco, Waltham, MA, USA) at 37 °C in 5% CO_2_. The medium was refreshed every 3 days. After reaching 80% confluence, the cells were detached with 0.25% trypsin/EDTA (Gibco, Waltham, MA, USA) and passaged.

#### Characterization of DPSCs

For phenotype analysis, third-generation DPSCs were digested, harvested, and washed with cold PBS. The cells were then labeled with PE-conjugated antibodies against CD105, CD44, CD90, CD19, and CD45, and with FITC-conjugated antibodies against CD29, HLA-DR, and CD34, (Abcam, Cambridge, UK) for 30 min in the dark at 4 °C. The cells were washed twice with stain buffer and analyzed using flow cytometry (Beckman Coulter, Placentia, CA, USA).

To evaluate the multilineage differentiation capacity, DPSCs were cultured in the corresponding differentiation medium after reaching 80% confluence. The osteogenic differentiation medium consisted of α-MEM supplemented with 10% FBS, 10 nM dexamethasone (Sigma-Aldrich, St Louis, MO, USA), 0.2 mM ascorbic acid (Sigma-Aldrich, St Louis, MO, USA), and 10 mM β-glycerophosphate disodium salt hydrate (Sigma-Aldrich, St Louis, MO, USA). The medium was refreshed every 3 days. After 21 days, alizarin red (Solarbio, Beijing, China) staining was used to detect the formation of mineralized nodules. The adipogenic differentiation medium consisted of α-MEM supplemented with 10% FBS, 0.5 mM 3-isobutyl-1-methylxanthine (Solarbio, Beijing, China), 2 μM dexamethasone, 0.2 mM indomethacin (Sigma-Aldrich, St Louis, MO, USA), and 0.01 g/L insulin (Sigma-Aldrich, St Louis, MO, USA). After 28 days, the cells were stained with oil-red O (Solarbio, Beijing, China). The chondrogenic differentiation medium (Cyagen Biosciences Inc., CA, USA) is according to the manufacturer’s instructions. After 28 days, the cells were stained with alcian blue (Solarbio, Beijing, China).

### Formation of the cell-sheets

To create cell sheets, DPSCs were seeded in tissue culture plates at a density of 2.5 × 10^4^ cells/cm^2^ and cultured for 7 days. The culture medium consisted of basal medium and 0.2 mM ascorbic acid. The culture medium was refreshed every 2–3 days. After 7 more days of culture, the DPSCs sheet was formed for further experiments.

### Isolation and characterization of DPSC-Exos

#### Isolation of DPSC-Exos

After DPSCs cell sheets were cultured in Umibio serum-free media (Umibio, Shanghai, China) for 48 h, the supernatants were collected and centrifuged at 300×*g* for 20 min, 2000×*g* for 20 min, and 10,000×*g* for 30 min to remove residual cells and debris. Then the supernatants were ultracentrifuged at 100,000×*g* for 17 h at 4 °C using ultracentrifugation by Optima XE-90 ultracentrifuge (Beckman Coulter, Placentia, CA, USA). The exosomes were resuspended in PBS and quantified using a Micro-BCA protein assay kit (Thermo Fisher Scientific, Waltham, MA, USA). The fractions were stored at − 80 °C until use.

#### Characterization of DPSC-Exos

The exosomes were characterized by transmission electron microscopy (TEM), and their morphology was observed. Nanoparticle tracking analysis (NTA) was used to analyze the size distribution of exosomes and the concentration of nanoparticles. Specific exosome markers (CD63, ALIX, and TSG101) were used as a positive control between DPSCs and the exosomes group for Western Blot.

### Exosomes labeling and tracking in vitro

A253 cells line purchased from ATCC were cultured in Roswell Park Memorial Institute (RPMI) 1640 (Gibco, Waltham, MA, USA) supplemented with 10% FBS (BI, USA) at 37 °C in 5% CO_2_ and used as an SGEC model [26]. To detect the uptake of exosomes by SGEC, exosomes were incubated with 1 μM PKH26 Fluorescent Cell Linker Kit (Sigma-Aldrich, MA, USA) for 10 min at 37 °C followed by centrifuged at 100,000×*g* for 70 min to remove excess dye. The labeled exosomes were then co-cultured with SGEC for 4 h. After treatment, cells were washed twice with PBS and fixed with 4% paraformaldehyde. Following DAPI staining, the cells were observed under a fluorescence microscope.

### Animal study

Female NOD/ltj mice (6-week-old, 20–22 g) were purchased from Cavens Laboratory Animal Co., Ltd. (Changzhou, China) and maintained in an SPF environment. All mice were free to obtain soy-based food and water under the light/dark cycle for 12/12 h at a constant temperature of 25 ± 1 °C. The Ethics Committee Board for the care and use of laboratory animals approved all the experiments in this study (HWT-BG-117).

Female NOD/Ltj mice served as SS animal models which were randomly divided into a treatment group (n = 6), a positive control group (hydroxychloroquine-treated) (n = 6), and a disease group (n = 6). For the treatment group, the mice were injected with DPSC-Exos (25 mg/kg) into the tail vein once a week for 10 weeks. Hydroxychloroquine (HCQ) gastric-infused (60 mg/kg) mice served as positive controls, and the NOD/ltj mice injected with an equal volume of PBS served as a negative control. The saliva secretion flow rates were recorded weekly after the 1st injection. After 10 weeks of drug administration, mice were sacrificed. Submandibular glands and venous blood of mice were used for other experiments.

### Measurement of saliva flow rates

After weighing the mice, they were anesthetized and injected with pilocarpine hydrochloride (0.1 mg/kg i.p.) to stimulate salivation. Saliva was collected 10 min after the pilocarpine injection as follows: a glass capillary was placed on the side of the mouth under the tongue and held steadily during a 10-min period to collect saliva into a tube. The weight difference in the tubes before and after saliva collection was calculated.

### Histology, immunohistochemistry, and immunofluorescence staining

After euthanizing mice, submandibular glands were collected and immediately fixed with 4% paraformaldehyde. Paraformaldehyde-fixed tissues were embedded in paraffin. Tissue sections (4 μm thick) were cut and stained with hematoxylin and eosin for morphologic examination.

The sections were dewaxed and rehydrated for immunohistochemistry with xylol and alcohol, respectively. Antigen retrieval was conducted by sodium citrate in microwave conditions. After then, 1% H_2_O_2_ was used to block endogenous peroxidase activity. The slides were further blocked with goat serum for 30 min. The antibodies of anti-AQP5 (1:200) and anti-GPER (1:200) were incubated with the slides at 4 °C overnight. The next day, after washing with PBS, the secondary antibody was added, and diaminobenzidine (DAB) was applied to visualize the image using a microscope (Olympus, Tokyo, Japan). The sections were dewaxed and rehydrated for immunofluorescence staining with xylol and alcohol, respectively. Then, the Specific steps were performed in the same operation as above in vitro.

### Cell treatment

SGEC were pretreated with 50 ng/ml IFN-γ (Peprotech, Offenbach, Germany) for 12 h to mimic SS inflammatory condition in the salivary gland. To investigate the effect of DPSC-Exos on SGEC viability and functions, the cultures were treated with DPSC-Exos (5, 20, or 80 μg/ml) for 48 h.

### Cell counting kit‑8 (CCK‑8) assay

SGEC were seeded into 96‑well plates (6 × 10^3^ cells/well) and pretreated with IFN‑γ and DPSC-Exos were added to cell culture subsequently. The cell proliferation was detected using a CCK-8 solution (Dojindo, Kumamoto, Japan) at 24, 48, and 72 h of culture according to the manufacturer’s instructions. Each test was repeated at least three times. (Thermo Fisher Scientific, Waltham, MA, USA).

### RNA isolation and real‐time quantitative PCR (RT-qPCR) analysis

The total RNA from SGEC and submandibular glands from NOD/ltj mice were extracted by a total RNA extraction kit (Accurate Biotechnology, Hunan, China). Two micrograms of total RNA were used to synthesize cDNA (Accurate Biotechnology, Hunan, China). RT-qPCR was performed using an SYBR Green RT-qPCR kit (Accurate Biotechnology, Hunan, China). Relative mRNA expression was normalized to that of the internal GAPDH control. The primer sequences are listed in Table 1. The relative expression of targeted genes was calculated by the 2^−△△Ct^ method. Each test was repeated at least three times.

**Table 1:** Primer sequences

Genes	Acc. no.	Reverse primers (5′–3′)	Product length (bp)
hGAPDH	NM_001357943.2	F: GGACCTGACCTGCCGTCTAG R: GTAGCCCAGGATGCCCTTGA	100
hAQP5	NM_001651.4	F: CGGGCTTTCTTCTACGTGG R: GCTGGAAGGTCAGAATCAGCTC	169
mGAPDH	NM_001289726.1	F: AAGAAGGTGGTGAAGCAGG R: GAAGGTGGAAGAGTGGGAGT	111
mAQP5	NM_009701.4	F: GCCCTCTTAATAGGCAACCAG R: GCATTGACGGCCAGGTTAC	140

### Western blot analysis

The total protein was extracted using RIPA lysis buffer (Thermo Fisher Scientific, Waltham, MA, USA) with protease inhibitor and phosphatase inhibitors (Beyotime, Nanjing, China) and quantified by a BCA protein assay kit (Thermo Fisher Scientific, Waltham, MA, USA). Total protein extracts (30 µg) from exosomes, cells, and submandibular gland tissue were separated via 10% SDS‑PAGE (Epizyme, Shanghai, China) and transferred to polyvinylidene fluoride membranes (Millipore, Burlington, MA, USA) and blocked with 5% non‑fat milk for 1 h at room temperature. After incubation with the primary antibody and the secondary antibody, the target protein was visualized by chemiluminescence using an ECL kit (Beyotime, Nanjing, China). The antibodies used in the Western blot assay are listed as follows: GAPDH (1:10,000, ab181602, Abcam), AQP5 (1:1000, sc-514022, Santa Cruz), CD63 (1:1000, ab134045, Abcam), TSG101 (1:1000, ab125011, Abcam), ALIX (1:1000, ab275377, Abcam), GPER (1:1000, ab260033, Abcam), CREB (1:1000, ab32515, Abcam), p-CREB (1:5000, ab32096, Abcam), PKA (1:1000, #4782, Cell Signaling Technology), p-PKA (1:1000, #4781, Cell Signaling Technology) and goat anti-rabbit IgG H&L (HRP) antibody (1:5000, #31460, Thermo Fisher Scientific).

### Immunofluorescence staining

Cell slides and submandibular gland tissue samples were fixed with 4% paraformaldehyde for 20 min, permeabilized in 0.2% Triton X-100 for 20 min, and blocked with 1% bovine serum albumin (BSA) for 1 h. Then the cell slides and submandibular gland tissue samples were incubated with AQP5 antibodies (1:200) at 4 °C overnight, followed by incubation with fluorophore-conjugated secondary antibodies (1:400, SA00013-3, Proteintech) for 1 h at room temperature. DAPI staining for 10 min was carried out after secondary antibody incubation.

To verify whether G protein-coupled estrogen receptor (GPER) was involved in the regulation of salivary secretion in vitro, cell slides were performed the same operation as above and then were incubated with GPER antibodies (1:200) at 4 °C overnight, followed by incubation with fluorophore-conjugated secondary antibodies (1:200, SA00013-2, Proteintech) for 1 h at room temperature. DAPI staining for 10 min was carried out after secondary antibody incubation. Staining was detected using fluorescent microscopy (Olympus, Tokyo, Japan).

### mRNA‐sequencing

The total RNA of IFN‑γ induced SGEC treated for 48 h with or without DPSCs-Exos was isolated using the TRIzol reagent (Invitrogen Life Technologies, USA) following the manufacturer’s protocol. RNA concentration and purity were checked with a Nanodrop 2000 instrument (Thermo Fisher Scientific, USA). After RNA quality control was performed, the libraries for next-generation sequencing were prepared using the TruSeqTM RNA Sample Prep Kit (Illumina, USA) according to the manufacturer’s instructions. Sequencing was performed by Shanghai Origingene Biopharm Technology Co., Ltd. (Shanghai, China). After quality control of the original data, the high-quality sequencing data were compared with the designated reference genome.

### Bioinformatic analysis

Raw data were processed using the robust multiarray mean (RMA) algorithm in the “Affy” package in the R language (http://cran.r-project.org/), including background correction, normalization, and probe summarization. Statistically significant differentially expressed genes (DEGs) were mined using linear models from the “LIMMA” package in R language. Volcano plots were generated using the “ggplot2” package in the R language. “P-value < 0.05 and |log2FC| > 1” were critical values for screening DEGs. Pearson’s correlation test verified the reproducibility of data within each group. The within-group data repeatability of the dataset was tested by sample cluster analysis. Statistical analysis was performed using the R language, and the “ggplot2” package presented the results. DAVID (http://david.abcc.ncifcrf.gov/) online tool was used for classifying gene function and evaluating the biological function of genes. Gene ontology (GO) and Kyoto Encyclopedia of Genes and Genomes (KEGG) enrichment analysis were performed using the DAVID database to study the role of DEGs. P < 0.05 was taken as a statistically significant cut-off point. To investigate the protein–protein interaction (PPI) network, we used the STRING (v11.5, https://string-db.org) and visualized it and analyzed the interactions of salivary secretion, cAMP signaling pathway, and estrogen signaling pathway by the Cytoscape (v3.8.2, https://cytoscape.org) software.

### Enzyme-linked immunosorbent assay (ELISA)

Venous blood was taken from the heart of mice and the serum was obtained by centrifugation. The levels of anti-SSA/Ro and anti-SSB/La autoantibodies were detected by mice anti-SSA/Ro and anti-SSB/La ELISA kit (Mlbio, Shanghai, China) according to the manufacturer’s protocol.

Cell lysates were prepared from in vitro culture of SGEC using RIPA lysis buffer (Thermo Fisher Scientific, Waltham, MA, USA). The level of cyclic AMP (cAMP) in cell lysate was analyzed by a human cAMP ELISA kit (Mlbio, Shanghai, China). The optical density was detected at 450 nm by a microplate reader.

### ***Measurement of intracellular Ca***^***2***+^***levels***

Intracellular Ca^2+^ levels were measured by Fluo-2 AM (Beyotime, Nanjing, China) staining kit according to the manufacturer’s instructions. Briefly, SGEC were cultured in 6-well black plates (4 × 10^5^ cells/well). After 48 h, cells were trypsinized and incubated with Fluo-2 AM for 30 min at 37 °C. The concentration of Fluo-2 was detected by flow cytometry.

### Statistical analysis

The data are expressed as mean ± standard deviation (SD). Statistical analysis was performed with t-tests for the comparison of two groups and a one-way analysis of variance with Tukey’s posthoc test for multiple group comparison. p value < 0.05 was considered statistically significant. GraphPad Prism 7.0 (GraphPad Software, CA, USA) was used for statistical analysis and bar figure preparation.

## Results

### Isolation, culture, and identification of DPSCs

**Fig. 1:**
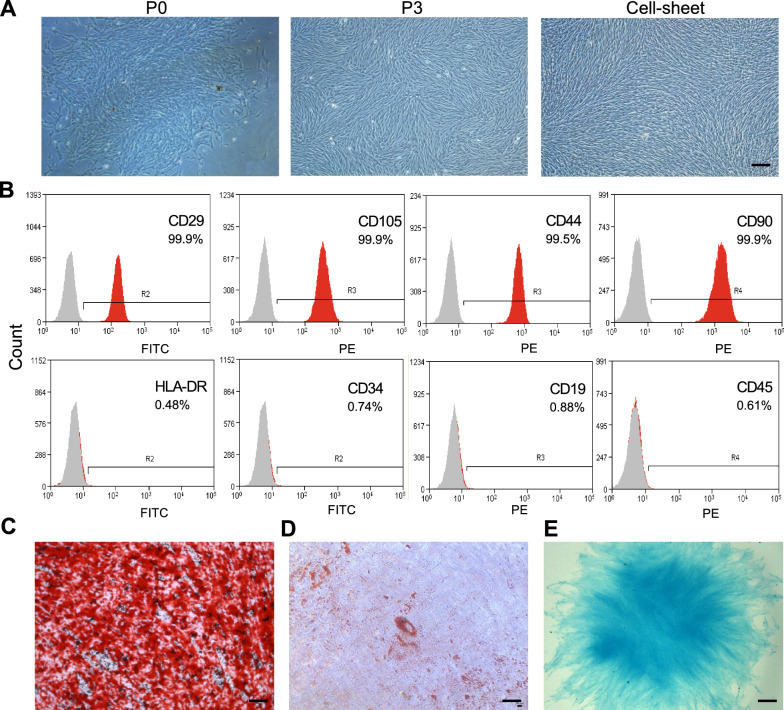
Isolation and characterization of DPSCs. **A** Human DPSCs culture at primary passage (P0), passage 3 (P3), and cell sheet. Scale bar: 200 μm. **B** Analysis of DPSCs surface markers by flow cytometry. **C** Alizarin red, **D** oil red O, and **E** alcian blue staining of DPSCs after inducing osteogenic, adipogenic, and chondrogenic differentiation respectively. Scale bar: 100 μm

Human DPSCs were isolated and observed under an inverted phase contrast microscope during the different passages of cultures and cell-sheet cultures (Fig. 1A). DPSCs showed fibroblast-like cell morphology. DPSCs highly expressed MSCs surface markers CD29 (99.9%), CD90 (99.9%), CD105 (99.9%), and CD44 (99.5%). Hematopoietic cell markers CD34, CD45, CD19, and HLA-DR expression were < 1% (Fig. 1B), indicating low immunogenicity of DPSCs [27]. Multilineage differentiation assays showed the osteogenic (Fig. 1C), adipogenic (Fig. 1D), and chondrogenic differentiation (Fig. 1E) potential of DPSCs. Our results confirm the MSC phenotype of DPSCs used in this study.

### Isolation and identification of DPSC-Exos

**Fig. 2:**
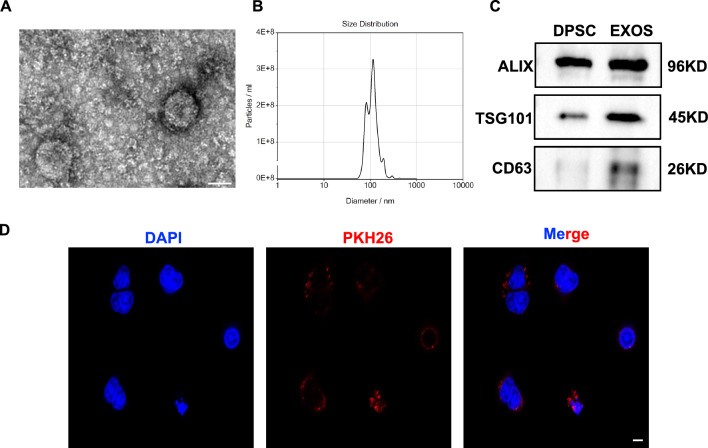
Isolation and characterization of DPSCs cell sheet released exosomes-released exosomes (DPSC-Exos). **A** Representative TEM images of DPSC-Exos. Scale bar: 100 nm. **B** Nanoparticle tracking analysis of DPSC-Exos. **C** Western blot analysis of exosome-specific markers ALIX, TSG101, and CD6. **D** Immunofluorescence staining for DPSC-Exos uptake by salivary gland epithelial cells (SGEC). Scale bar: 20 μm

DPSC-Exos was isolated from DPSCs cell sheet culture-conditioned medium by ultracentrifugation. TEM micrographs revealed that DPSC-Exos displays a bilayer membrane structure with typical cup-shaped morphology (Fig. 2A). NTA showed a size distribution of DPSC-Exos from 30 to 150 nm (Fig. 2B). DPSC-Exos expressed typical exosome markers ALIX, TSG101, and CD63 (Fig. 2C). DPSC-Exos were easily internalized in SGEC within 4 h of co-culture (Fig. 2D). Together, these data indicate the successful isolation of DPSC-Exos that can be internalized in SGEC.

### DPSC-Exos partially rescued IFN-γ-induced SGEC death and downregulation of AQP5

**Fig. 3:**
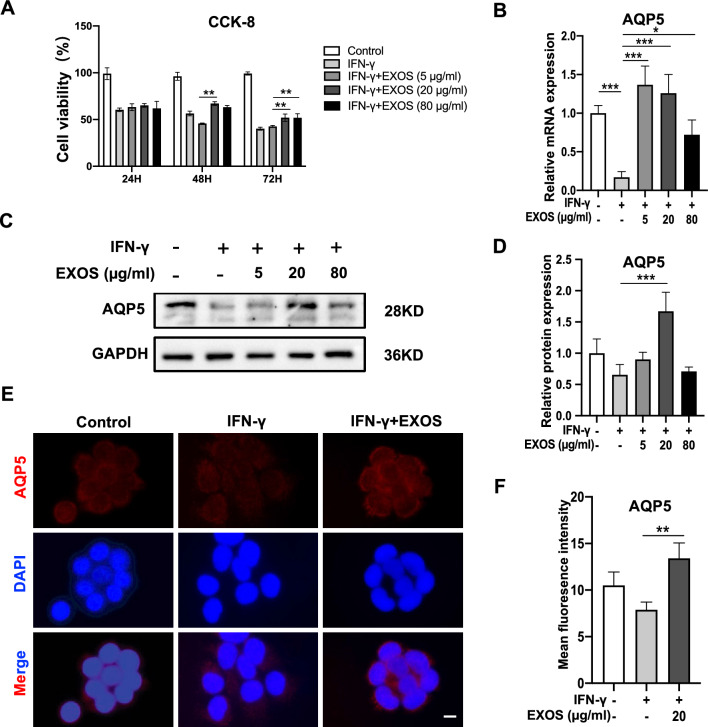
DPSC-Exos rescued IFN-γ-caused death and AQP5 downregulation of salivary gland epithelial cells (SGEC). **A** The cell viability of salivary gland epithelial cells was detected by CCK-8 assay. The expression pattern of AQP5 analyzed by RT-qPCR (**B**), Western blot analysis (**C**, **D**), and immunofluorescence staining (**E**, **F**). Scale bar: 20 μm. Data are presented as the mean ± SD, n = 3. Significant effect of the treatment, *p < 0.05, **p < 0.01, ***p < 0.001

IFN-γ secreted by infiltrating lymphocytes induces ductal apoptosis associated with SS, which is responsible for impairing gland secretory function [28]. Therefore, IFN-γ is commonly used in vitro to mimic inflammatory conditions in the salivary gland during SS [29]. In this study, IFN-γ treatment for 12 h dramatically inhibited SGEC proliferation (Fig. 3A). To verify the effect of DPSC-Exos on IFN-γ-induced death in SEGC, different concentrations of DPSC-Exos (5, 20, and 80 μg/ml) were added in SGEC for 24, 48, and 72 h. Compared with the IFN-γ group, the proliferation of the 20 and 80 μg/ml DPSC-Exos treatment group gradually increased at 48 and 72 h (Fig. 3A). These results indicate that DPSC-Exos have the potential to alleviate inflammation-inhibited SGEC proliferation during SS.

To monitor the changes in the endogenous AQP5 transcription level, we performed the RT-qPCR in SGEC after treatment of different concentrations of DPSC-Exos for 48 h. AQP5 mRNA relative expression was significantly decreased in the IFN-γ group. DPSC-Exos rescued the inhibitory effect of IFN-γ on AQP5 expression (Fig. 3B). In Western blot analysis, DPSC-Exos (20 μg/ml) treated group showed higher expression of AQP5 compared to the IFN-γ-treated group (Fig. 3C, D). In immunofluorescence analysis, the fluorescence intensity of AQP5 significantly increased in DPSC-Exos (20 μg/ml) group compared with the IFN-γ-treated group (Fig. 3E, F). These data indicate that DPSC-Exos have the potential to upregulate AQP5 expression in SGEC during SS.

### Transcriptome analysis of IFN-γ-pretreated SGEC with or without DPSC-Exos

**Fig. 4:**
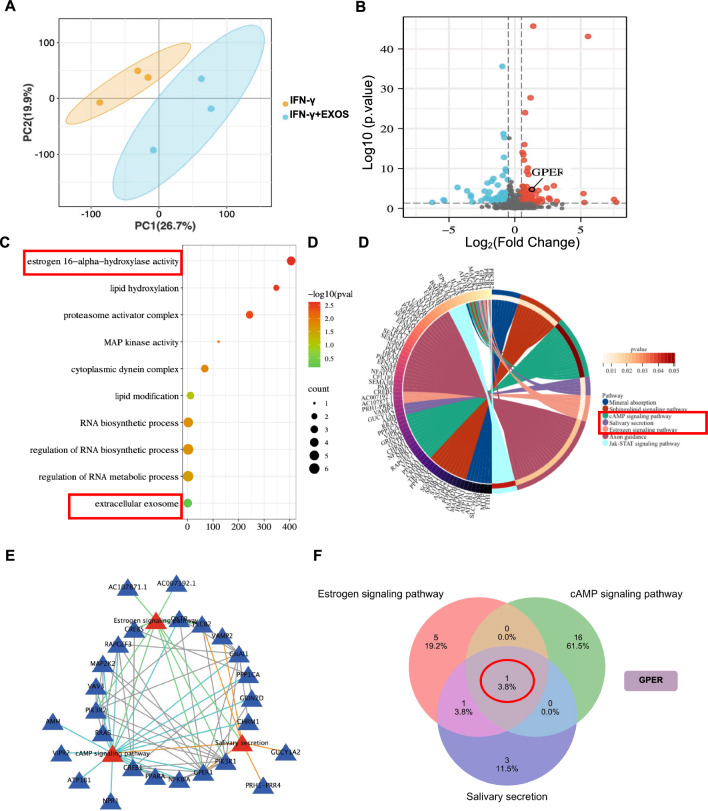
Transcriptome analysis of salivary gland epithelial cells treated with or without DPSC-Exos. **A** The Principal Component Analysis (PCA). **B** Volcano plot showing differentially expressed genes (DEGs). **C** Gene ontology analysis. **D** KEGG analysis enriched GPER-related pathways among DEGs. **E** STRING database shows the interaction among the cAMP signaling pathway, salivary secretion, and estrogen signaling pathway, and **F** Venn analysis of the genes among the cAMP signaling pathway, salivary secretion, and estrogen signaling pathway. All DEGs are screened based on P-value < 0.05 and |fold change| > 1

RNA sequencing was performed with and without DPSC-Exos (20 μg/ml) in IFN-γ-pretreated SGEC to identify differentially expressed genes (DEGs). Firstly, principal component analysis (PCA) showed data independence between the groups, indicating the comparability of data (Fig. 4A). A total of 304 DEGs were found with 142 upregulated and 162 downregulated in the DPSC-Exos group compared with the IFN-γ group. The GPER is one of the upregulated DEGs in the DPSC-Exos group (Fig. 4B), that plays role in estrogen 16-alpha-hydroxylase activity in SGEC according to the Gene Ontology (GO) enrichment analysis (Fig. 4C), and cAMP signaling pathway, salivary secretion, and estrogen signaling pathway according to Kyoto Encyclopedia of Genes and Genomes (KEGG) analysis (Fig. 4D). Analysis of interactive relationships of DEGs and the STRING database showed the interactions of salivary secretion, cAMP signaling pathway, and estrogen signaling pathway (Fig. 4E). Moreover, the overlap analysis showed GPER as a key DEG involved in the interaction of these 3 signaling pathways (Fig. 4F). These data indicate a possible role of overexpressed GPER of the DPSC-Exos group in the improved SGEC activity.

### DPSC-Exos group showed a higher expression of GPER

**Fig. 5:**
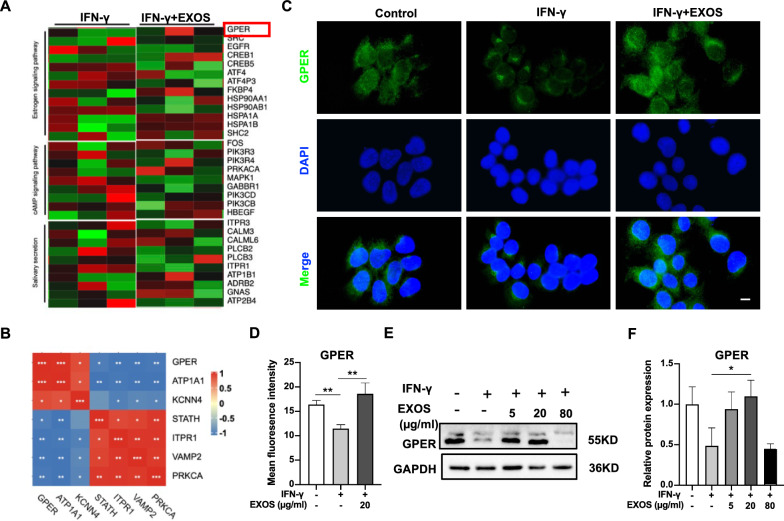
DPSC-Exos treatment induced GPER expression in salivary gland epithelial cells (SGEC). **A** Heatmap of differentially expressed genes (DEGs) related to cAMP signaling pathway, salivary secretion, and estrogen signaling pathway. **B** Correlation heatmap between GPER and the marker of salivary secretion in DEGs. **C**, **D** Immunofluorescence staining for GPER expression in SGEC. **E**, **F** Western blot analysis of GPER expression in SGEC. Scale bar: 20 μm. Data are presented as the mean ± SD, n = 3. Significant effect of the treatment, *p < 0.05, **p < 0.01, ***p < 0.001

The heat map showed upregulated GPER in DPSC-Exos-treated SGEC (Fig. 5A). The correlation heatmap indicated that GPER is closely associated with DEGs related to salivary secretion (Fig. 5B). Immunofluorescence analysis showed downregulation of GPER in the IFN-γ group and DPSC-Exos rescued IFN-γ-inhibited GPER expression in SGEC (Fig. 5C, D). Western blot analysis confirmed the higher expression of GPER in the DPSC-Exos group compared with the IFN-γ group (Fig. 5E, F). These data demonstrate that DPSC-Exos have the potential to upregulate GPER expression in IFN-γ-treated SGEC.

### DPSC-Exos treatment alleviated xerostomia and revitalized salivary gland function in NOD/ltj mice

**Fig. 6:**
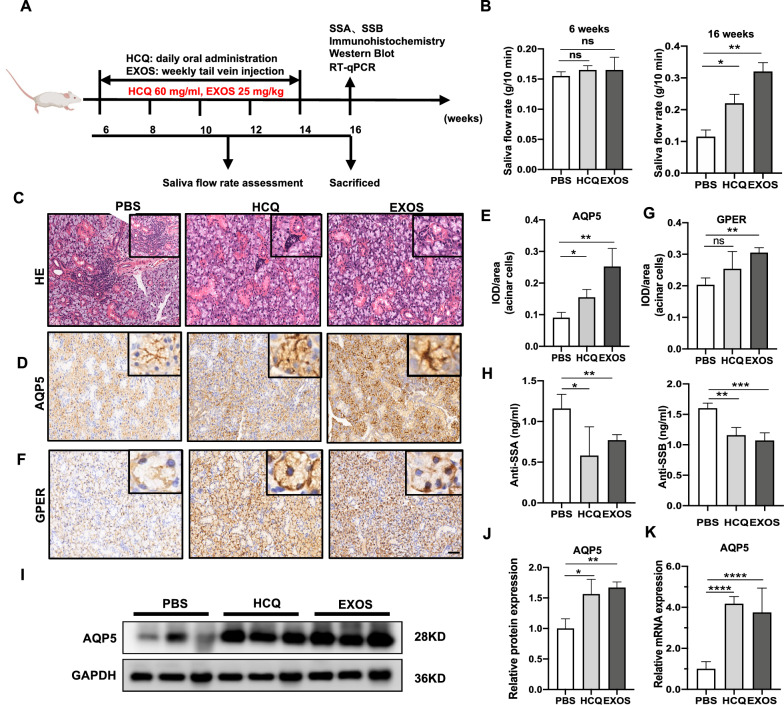
DPSC-Exos treatment alleviated SS-like symptoms in NOD/ltj mice. **A** Scheme of animal experiment and treatment. **B** The saliva flow rate, **C** H&E stained histological images of mice submandibular glands. Immunohistochemistry analysis of AQP5 (**D**, **E**) and GPER (**F**, **G**) expression in mice submandibular glands. **H** The anti-SSA/SSB levels of NOD/ltj mice serum were detected by ELISA. Western blot (**I**, **J**) and RT-qPCR analysis (**K**) of AQP5 expression in mice submandibular. Scale bar: 100 μm. Data are presented as the mean ± SD, n = 6. Significant effect of the treatment, *p < 0.05, **p < 0.01, ***p < 0.001

NOD/ltj mice were used as a primary SS model, which uniquely exhibits salivary gland dysfunction concomitant with the appearance of leukocyte infiltrations in the exocrine glands and the many congenic strains with known genetic differences [30]. The animal study was performed as illustrated in (Fig. 6A). The saliva flow rate was reduced in 16-week NOD/ltj mice than in 6-week NOD/ltj mice. Ten weeks of DPSC-Exos treatment robustly promoted the saliva flow rate in NOD/ltj mice (Fig. 6B). The effect of DPSC-Exos treatment on saliva flow rate was even more pronounced than the HCQ treatment (positive control). As indicated by H&E staining, the vacuolation of the acini around the glandular ducts became significant, and the glands were severely atrophied in the NOD/ltj mice. The number and area of lymphocyte infiltration foci in salivary glands were considerably reduced in HCQ and DPSC-Exos-treated NOD/ltj mice than those in PBS-treated NOD/ltj mice (Fig. 6C). Based on the fact that DPSC-Exos ameliorate the impairment of saliva secretion in NOD/ltj mice, we next investigated the expression of the critical protein AQP5 and GPER involved in saliva secretion in the submandibular glands. Immunohistochemistry (Fig. 6D, E) and Immunofluorescence staining (Additional file 1: Fig. S1A, B) showed that the AQP5 immunostaining at the membrane of acinar cells, especially at the apical membrane, was strongly enhanced in the DPSC-Exos group compared with the PBS group. GPER expression was also upregulated in the DPSC-Exos group compared with the PBS group (Fig. 6F, G). In contrast, GPER was frequently colored in the basement membrane, distinguishing it from AQP5 immunostaining. Anti-SSA/Ro and anti-SSB/La antibodies are associated with a higher incidence in SS patients. Therefore, it is of great value for the clinical diagnosis of SS [31]. DPSC-Exos and HCQ treatment downregulated the anti-SSA/Ro and anti-SSB/La serum levels in NOD/ltj mice (Fig. 6H). We performed the Western blot assay (Fig. 6I, J) and RT-qPCR assay (Fig. 6K) to detect the AQP5 expression level in the mice’s submandibular glands. The mRNA and protein level expressions of AQP5 in the DPSC-Exos and HCQ group were upregulated compared with the PBS group. These data indicated that DPSC-Exos upregulates the expression of AQP5 and GPER in the submandibular glands.

### *DPSC-Exos upregulated AQP5 in SGEC *via* GPER-mediated cAMP/PKA/CREB signaling pathway*

**Fig. 7:**
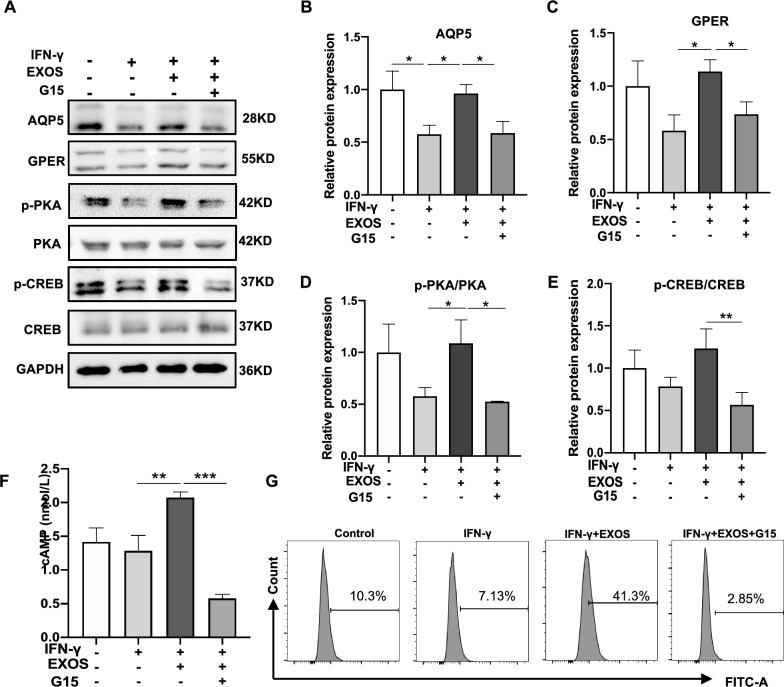
DPSC-Exos alleviated IFN-γ-caused AQP5 downregulation in salivary gland epithelial cells (SGEC) via GPER mediated cAMP-PKA-CREB pathway. **A**–**E** Western blot analysis AQP5, GPER, p-PKA, and p-CREB in SGEC. **F** The cAMP concentration in SGEC lysate was detected by ELISA. **G** The intracellular Ca^2+^ level of SGEC was measured by flow cytometry. Data are presented as the mean ± SD, n = 3. Significant effect of the treatment, *p < 0.05, **p < 0.01, ***p < 0.001

The activation of GPER stimulates the production of cAMP and intracellular calcium mobilization [32]. We analyzed the expression of 4 significant proteins in the cAMP/PKA/CREB signaling pathway AQP5, GPER, PKA (p-PKA), and CREB (p-CREB) in SGEC with different treatment conditions. DPSC-Exos group showed higher expression of AQP5, GPER, PKA (p-PKA), and CREB (p-CREB) compared with the IFN-γ group (Fig. 7A–E). This effect was nullified by the treatment of G15 (GPER inhibitor). The result of the cAMP level in SGEC with different treatment conditions was consistent with the result of Western blot analysis (Fig. 7F). AQP5 is in lipid rafts under unstimulated conditions and can move to the apical plasma membrane when intracellular calcium concentration rises [33, 34]. Therefore, the calcium signal is the core signal of AQP5 short-term regulation. The result of flow cytometry showed that the level of intracellular Ca^2+^ in the DPSC-Exos group was about fourfold higher compared to the IFN-γ group, while G15 treatment nullified the effect of DPSC-Exos on the level of intracellular Ca^2+^ (Fig. 7G). These results indicated that DPSC-Exos rescues the IFN-γ-inhibited function of SGEC via GPER-mediated activation of the cAMP/PKA/CREB signaling pathway.

## Discussion

**Fig. 8:**
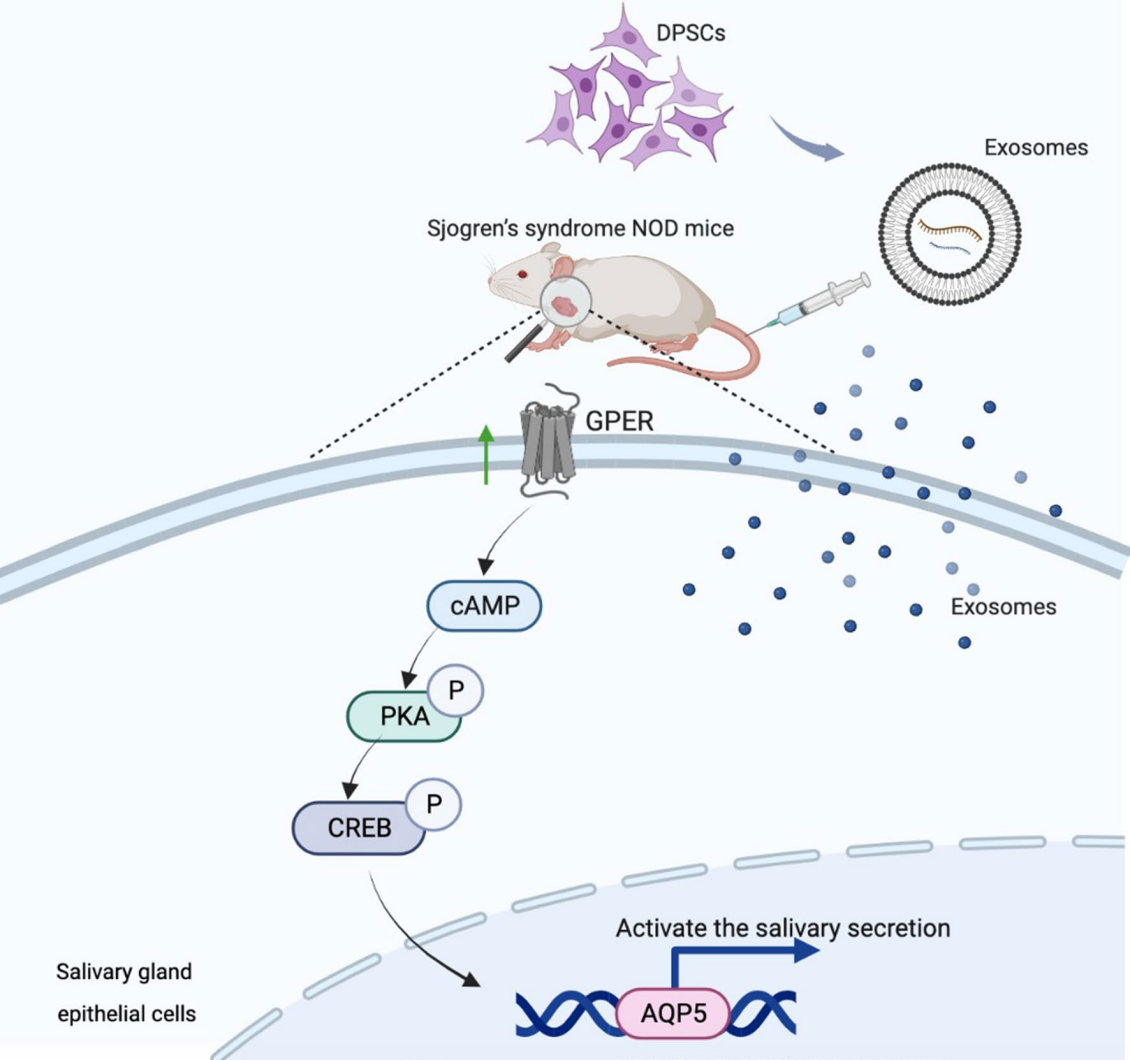
Schematic diagram of DPSC-Exos-mediated activation of saliva secretory function in NOD/ltj mice

SS is an autoimmune disease characterized by the infiltration of immune cells in exocrine glands and impaired exocrine gland secretory function. Commonly prescribed immunotherapies for SS only modulate immune cell function but fail to restore the salivary gland secretion function [35]. This study found that DPSC-Exos restore SS-impaired SGEC survival and function in vivo and in vitro models. DPSC-Exos upregulated GPER expression in SS SGEC to restore saliva secretion via activation of the cAMP/PKA/CREB pathway. Our results indicate the possible therapeutic potential of DPSC-Exos for SS (Fig. 8).

Exosomes are a type of nano-scaled vesicles with sufficient signaling substances which are secreted and released by living cells [36]. In shreds of literature, exosomes have been isolated from MSCs for various biomedical applications [37, 38]. In this study, DPSC-Exos isolated by ultracentrifugation method showed a size of 30–150 nm and expressed MSC exosome markers TSG101, ALIX, and CD63, which is in accordance with the characterization of DPSC-Exos in previous studies [11, 39]. Currently, MSC-Exos have been applied to treat various autoimmune diseases such as rheumatoid arthritis (RA), systemic lupus erythematosus (SLE), and systemic sclerosis (SSc) [10]. Considering these facts, we tested the efficacy of DPSC-Exos in improving the function of SGEC during SS. AQP5 is a key secretory protein that is downregulated in SGEC during SS [40]. IFN-γ is the inflammatory cytokine released by infiltrated immune cells in the salivary gland that mainly disrupts SGEC survival and functions [41]. Therefore, IFN-γ is commonly used to induce SS inflammatory conditions in SGEC in vitro [29]. In this study, DPSC-Exos partially rescued the IFN-γ-induced SGEC death. DPSC-Exos also upregulated AQP5 expression in IFN-γ-treated SGEC. Moreover, DPSC-Exos treatment increased the salivary flow rate and AQP5 expression in the salivary gland of SS model NOD/ltj mice. In this study, 20 μg/ml of DPSC-Exos was more effective against IFN-γ-induced effects on SGEC compared with 80 μg/ml DPSC-Exos. Highly pure exosome isolation is still a challenge and ultracentrifugation based exosome isolation contians impurities of damaged exosomes [42]. The impurities in exosomes lead to compromised therapeutic effect, which might be cause of the compromised therapeutic effect of 80 μg/ml DPSC-Exos. Shreds of literature, including our own study, indicate that upregulation of AQP5 in SGEC could alleviate xerostomia in SS [24]. This is the first study to show that DPSC-Exos treatment in SS upregulates AQP5 expression in SGEC.

GPER is a transmembrane estrogen receptor, connecting with 17β-estradiol [32]. Traditionally, 17β-estradiol mediates rapid signaling events via pathways that involve GPER. For decades, the potential role of GPER has been elucidated connecting with various diseases such as reproductive, endocrine, and immune systems. In this study, DPSC-Exos upregulated GPER expression in IFN-γ-treated SGEC and salivary glands of NOD mice. KEGG-analysis of transcriptome data indicated activation of estrogen and cAMP signaling, and salivary secretion in the DPSC-Exos+IFN-γ-treated SGEC compared with the IFN-γ treated SGEC. GPER stimulates cAMP production and calcium mobilization. The role of the cAMP-PKA/CREB pathway in the regulation of AQP5 production in rat nasal epithelium had been reported [42]. Also, Ca^2+^ signaling is a principal signal in both protein and water secretion from salivary glands induced by cholinergic stimulation [43]. Inhibition of GPER reversed the DPSC-Exos-induced upregulation of AQP5, p-PKA, p-CREB, cAMP, and intracellular mobilization of Ca^2+^ ions in IFN-γ-pretreated SGEC. Our results elucidated GPER mediated cAMP-PKA-CREB pathway as the mechanism of DPSC-Exos-mediated alleviation of xerostomia in SS.

Although the exact pathogenic mechanisms of the autoimmune responses in SS are not fully understood, epithelial cells appear to play a key role in autoimmune responses in SS [44]. In this study, DPSC-Exos treatment inhibited the anti-SSA/Ro and anti-SSB/La serum levels in NOD/ltj mice. These results indicate that DPSC-Exos has the potential to inhibit the auto-immune responses in SS. Various studies have shown the immunomodulatory and anti-inflammatory role of MSC-Exos [45–47]. In this study, the DPSC-Exos were applied systemically in NOD/ltj mice. Therefore, the possible effect of DPSC-Exos on immune modulation and inflammation inhibition in NOD/ltj mice should be thoroughly investigated in future studies. This is the first study to report the therapeutic effects of DPSC-Exos on SS via GPER-mediated activation of the cAMP-PKA-CREB pathway in SGEC. However, the exact mechanism of DPSC-Exos-mediated GPER upregulation in SGEC should be further investigated.

## Conclusion

DPSC-Exos partially rescued SS-related inflammation-induced death of SGEC. SS-related inflammation decreased salivary secretion marker AQP5 expression in SGEC and DPSC-Exos reversed this effect. DPSC-Exos treatment alleviated glandular inflammation and increased the saliva flow rate in NOD/ltj mice. DPSC-Exos treatment upregulated GPER in SGEC which further activated cAMP-PKA-CREB signaling to promote salivary secretion. Our results indicate the possible application of DPSC-Exos on SS treatment.

## Supplementary Information

**Additional file 1: Figure S1.** DPSC-Exos treatment in vivo enhanced AQP5 expression.Immunofluorescence staining of AQP5 in mice submandibular glands. Scale bar: 100 μm. Data are presented as the mean ± SD, n = 6. Significant effect of the treatment, *p < 0.05, **p < 0.01, ***p < 0.001.

## Data Availability

The datasets used and/or analyzed during the current study are available from the corresponding author on reasonable request.

## Abbreviations

SS: Sjogren’s syndrome
MSCs: Mesenchymal stem cells
DPSCs: Dental pulp stem cells
DPSC-Exos: Dental pulp stem cells derived exosomes
IFN-γ: Interferon-gamma
SGEC: Salivary gland epithelial cells
DEGs: Differentially expressed genes
NOD: Non-obese diabetic
GO: Gene ontology
KEGG: Kyoto Encyclopedia of Genes and Genomes
AQP5: Aquaporin 5
GPER: G-protein coupled estrogen receptor
CCK-8: Cell Counting Kit‑8
HCQ: Hydroxychloroquine
cAMP: Cyclic AMP
RA: Rheumatoid arthritis
SLE: Systemic lupus erythematosus
SSc: Systemic sclerosis
