# Supplementary material for: Dental pulp stem cell-derived exosomes revitalize salivary gland epithelial cell function in NOD mice via the GPER-mediated cAMP/PKA/CREB signaling pathway
Source: J Transl Med. 2023 Jun 3;21:361. doi: 10.1186/s12967-023-04198-0 (PMC10239098; doi:10.1186/s12967-023-04198-0)
Supplement: Supplementary file 1 — Additional file 1: Figure S1. DPSC-Exos treatment in vivo enhanced AQP5 expression.Immunofluorescence staining of AQP5 in mice submandibular glands. Scale bar: 100 μm. Data are presented as the mean ± SD, n = 6. Significant effect of the treatment, *p < 0.05, **p < 0.01, ***p < 0.001. [file 12967_2023_4198_MOESM1_ESM.pdf]

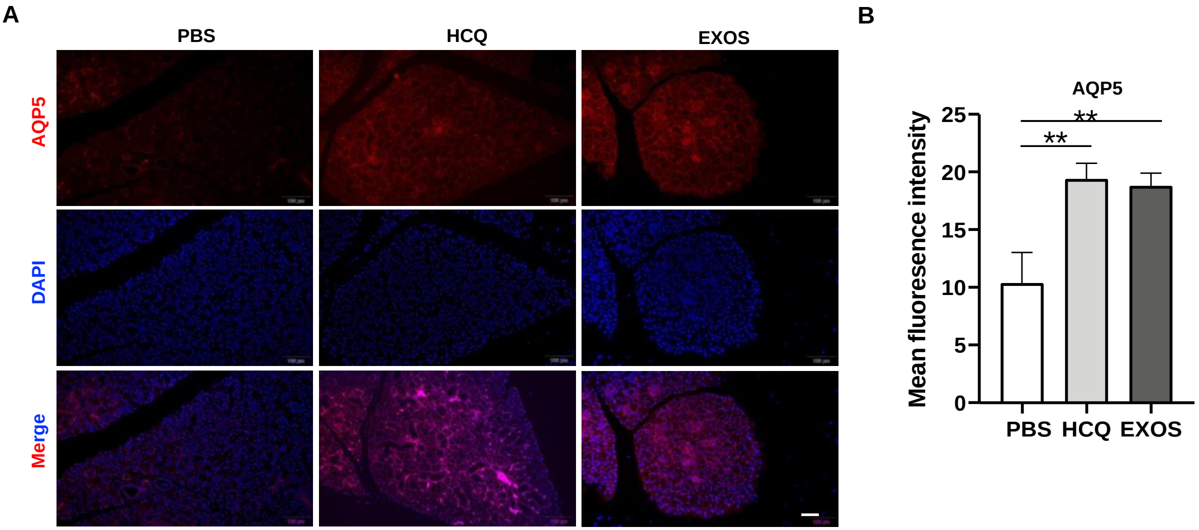

Figure S1. DPSC-Exos treatment in vivo enhanced AQP5 expression. (A, B) Immunofluorescence staining of AQP5 in mice submandibular glands. Scale bar: 100µm. Data are presented as the mean ± SD, n = 6. Significant effect of the treatment, *p < 0.05, **p < 0.01, ***p < 0.001.
